# Well-being and job satisfaction : a study of healthcare workers at Charles Nicole Hospital in Tunis

**DOI:** 10.1192/j.eurpsy.2025.1565

**Published:** 2025-08-26

**Authors:** F. E. Boujmil, M. Mersni, G. Bahri, S. Chemingui, D. Brahim, N. Ladhari, I. Besbes, A. Hakiri, R. Ghachem

**Affiliations:** 1 Occupational Health, Charles Nicole Hospital, Tunis; 2 Psychiatry - B, Razi Hospital, Manouba, Tunisia

## Abstract

**Introduction:**

Job satisfaction is undoubtedly one of the concerns that has given rise to the most research since the middle of the 20th century. It is one of the particularly important concepts in the study of ‘health at work’ determinants. Its evaluation may appear to be necessary in order to set up relevant preventive actions. However, in Tunisian hospitals, job satisfaction remains poorly assessed. It is therefore crucial to assess it and study its factors in order to promote a balanced work environment.

**Objectives:**

Assess the overall job satisfaction of health care workers (HCW) at Charles Nicole Hospital using a validated questionnaire in French and Arabic.

**Methods:**

This was a cross-sectional study of consenting HCW at Charles Nicole Hospital during the period from 1st September to 31st October 2024. Data were collected by means of a 63-item questionnaire, which assessed participants’ well-being and satisfaction on several aspects using a four-point Likert scale with six dimensions: Perceived superior support at work, affective commitment, emotional exhaustion, work/family conflict, extrinsic efforts and job satisfaction. The questionnaire had good internal consistency and was validated in French and Arabic. The questionnaire was self-administered to the HCW who went to the occupational health department for a medical check-up.

**Results:**

A total of 30 participants were enrolled. The mean age was 45.37 ± 10.3 years, with a sex ratio of 2.3 (F/H). Eighty-six percent were married and had at least one child. The most common occupational category was senior technician (34%). The average length of service was 17.27 ± 10.1 years. Laboratory departments and surgical departments were the most represented in 32% and 26% of cases respectively. Eighty percent of HCW worked full-time. Teamwork was reported in 53% of cases. The number of staff in the work team was less than 10 in 83.4% of cases. Perceived support from a superior was found in 40% of cases. Nearly 20% of cases showed emotional exhaustion secondary to their work and 34% showed extrinsic strain. Affective commitment was present in 36.7% of cases. Only 14.3% of cases showed work/family conflict. Overall, 73.3% of HCW were satisfied with their work.

**Conclusions:**

This study highlights the importance of working conditions and organisational support in the well-being of HCW. Job satisfaction, although mainly present, is put to the test by a mismatch between professional and personal life, extrinsic efforts and a lack of hierarchical support. Emotional exhaustion is a major warning sign, showing that better care and support could improve both emotional commitment and job satisfaction.

**Disclosure of Interest:**

None Declared

